# Vericiguat: A Promising Drug for the Treatment of Heart Failure

**DOI:** 10.2174/011573403X339474250320034144

**Published:** 2025-04-07

**Authors:** Drashti Shah, Alkesh Patel, Dharti Patel, Bhavesh Patel, Ashish Patel

**Affiliations:** 1 Department of Pharmaceutical Chemistry and Analysis, Ramanbhai Patel College of Pharmacy, Charotar University of Science and Technology, Charusat-Campus, Changa, 388421, Anand, Gujarat, India;; 2 Department of Pharmaceutical Chemistry, Faculty of Pharmacy, Institute of Pharmaceutical Sciences, Parul University, Vadodara, 391760, Gujarat, India;; 3 Department of Pharmaceutical Technology, Navinta LLC, Ewing Township, NJ 08618, USA;; 4 Department of Pharmaceutical Chemistry, Parul Institute of Pharmacy, Parul University, Vadodara, 391760, Gujarat, India

**Keywords:** Vericiguat, pyrazolo[3,4-b]pyridine, heart failure, soluble guanylate cyclase, cyclic guanosine monophosphate, oxidative stress

## Abstract

The health and survival of people with heart failure is a growing concern due to the associated illness and death. Traditional treatments such as medication, surgery, and lifestyle changes have not significantly improved life expectancy, leading to a search for more effective drug options. A drug that can act on oxidative stress and cardiac inflammatory markers while carrying the benefits of existing therapies is needed. Targeting the soluble guanylate cyclase (sGC)-cyclic guanosine monophosphate (cGMP) dependent pathway significantly reduces cardiac myocyte death and improves ejection fraction. In 2021, the USFDA approved Vericiguat, a derivative of pyrazolo[3,4-b]pyridine, to decrease the risk of cardiovascular death and hospitalization. This review provides information on the structure, pharmacokinetics, pharmacodynamics, clinical status, and treatment of Vericiguat in heart failure. Riociguat was the first sGC stimulator used in pulmonary hypertension therapy, but its short half-life required multiple dosing, making it unsuitable for cardiovascular diseases. Vericiguat was developed to address this limitation by decreasing metabolism, and both preclinical and clinical investigations have indicated its minimal pharmacokinetic interactions. This makes it appropriate for long-term use in cardiac patients with multiple comorbidities who require several medications. Vericiguat represents a promising new option for heart failure treatment, potentially improving patient outcomes and quality of life. Its compatibility with other heart failure therapies without significant drug-drug interactions further highlights its potential as a cornerstone treatment. Ongoing studies continue to explore its benefits, suggesting that vericiguat may enable more comprehensive and effective management of heart failure, reducing the burden of this debilitating condition.

## 1 INTRODUCTION

Heart Failure (HF), which prevents the heart from pumping sufficient blood to support the metabolism of the body, is a significant healthcare burden [1-4]. HF-related morbidity and mortality are still high despite advances in treatment and prevention, which emphasizes the need for more potent treatments [5, 6]. Currently, this disease affects more than 64 million people worldwide [7]. Despite recent improvements in HF therapy, the forecast intended for patients with symptomatic HF is still poor [8]. Additionally, studies conducted in the community indicate that up to 40% of patients die within a year of being diagnosed, and 60% to 70% die within five years, mainly because of heart failure that deteriorates and sudden cardiac death [9, 10]. In addition, HF places an annual financial pressure of about $108 billion on the entire world [1, 11].

Three subcategories of heart failure exist, including HF with preserved Ejection Fraction (HFpEF) where the Left Ventricular Ejection Fraction (LVEF) is 50% or higher, HF with mid-range ejection fraction (LVEF between 40-49%), and HF with reduced ejection fraction (HFrEF) where the LVEF is less than 40% [12, 13]. The presence and severity of HF are both indicated by the N-terminal pro-B-type natriuretic peptide (NT-proBNP) [14, 15] as an indicator of ventricular wall stress. The soluble Guanylate Cyclase (sGC) in the nitric oxide-sGC-cGMP pathway produces cyclic Guanosine Monophosphate (cGMP) to maintain normal cardiac and vascular function. This process is essential for the proper functioning of the cardiovascular system [16-18]. However, in HF, a deficit of cGMP occurs due to dysregulation of the nitric oxide-sGC-cGMP pathway.

Heart failure can be classified into four hemodynamic subtypes: warm + wet, cold + dry, cold + wet, and warm + dry, based on the presence or absence of clinical signs of hypoperfusion and congestion. This classification is crucial for guiding tailored therapeutic approaches. The warm + wet subtype represents patients with adequate perfusion but significant congestion, often presenting with symptoms such as raised jugular venous pressure, peripheral edema, pulmonary crackles, and dyspnoea due to fluid overload. The cold + dry subtype is characterized by hypoperfusion without congestion, with clinical signs including cold and vasoconstrictive extremities, agitation, confusion, hypotension, and fatigue. Patients in the cold + wet category experience both hypoperfusion and congestion, which is the most severe presentation, marked by cold extremities, oliguria or anuria, raised jugular venous pressure, pulmonary congestion, dyspnoea, and hypoxemia. Finally, the warm + dry subtype represents a compensated state with adequate perfusion and no congestion, where patients remain stable with normal vital signs, no dyspnoea, and the absence of peripheral edema. Understanding these subtypes allows for a practical framework for addressing the heterogeneity of heart failure and optimizing therapeutic strategies to improve patient outcomes [19, 20].

The first-in-class direct stimulator of sGC is vericiguat (CAS No: 1350653-20-1). It is used for the chronic HF (and Left Ventricular Ejection Fraction [LVEF] < 45%) therapy of hospitalized adult patients who are at risk of mortality due to cardiovascular risk [21-23]. The 15 mg oral tablet formulation of vericiguat is quickly engrossed in healthy volunteers and has a linear pharmacokinetic profile [24]. Two isoforms of Uridine diphosphate-Glucuronosyltransferase (UGT), UGT1A1 and UGT1A9, are expressed in the hepatic and renal tissues, respectively, involved in the metabolic pathway for vericiguat [25-27].

## 2 CHEMISTRY OF VERICIGUAT

Vericiguat is a derivative of 5-fluoro-1H-pyrazolo[3,4-b]pyridine, which has a 2-fluorobenzyl group substituted at the 1^st^ position and a 4,6-diamino-5-[(methoxycarbonyl) amino]pyrimidin-2-yl group substituted at the 3^rd^ position. These substitutions result in the formation of methyl N-[4,6-diamino-2-[5-fluoro-1-[(2-fluorophenyl)methyl]pyrazolo[3,4 -b]pyridin-3-yl]pyzimidin-5-yl]carbamate, as shown in Fig. (**1**).

Vericiguat has a molecular weight of 426.4 g/mol and a molecular formula of C_19_H_16_F_2_N_8_O_2_. This drug is considered to be highly permeable and is soluble in DMSO, but it is insoluble in ethanol and water. As a result, it is classified as a BCS class II drug. Vericiguat is a stimulator of sGC, and its activity is dependent on pH. Its predicted boiling point is 535.9°C. The route for the synthesis of vericiguat is illustrated in Fig. (**2**).

### 2.1 Synthesis of Vericiguat

Here, we reported some key steps for the synthesis of vericiguat. Firstly, the cyclization reaction occurs between ethyl-1-(2-fluorobenzyl)-5-amino-1H-pyrazole-3-carboxylate and (Z)-2-fluoro-3-morpholinoacryaldehyde to afford ester derivative of pyrazolo[3,4-b]pyridine (1). This intermediate (1) further reacts with sodium methoxide and formamide in the alcoholic mixture to get an amide derivative of pyrazolo[3,4-b]pyridine (2). In addition, this amide transforms into a nitrile group in phosphoryl chloride and in a mixture of sulfolane and acetonitrile to afford the nitrile derivative of pyrazolo[3,4-b]pyridine (3). In the fourth step, nitrile intermediate (3) reacts with ammonium chloride in sodium methoxide base to give amidine derivative (4) which further reacts with substituted azobenzene derivative by SN_2_ and reduction reaction in Triethylamine (TEA) base and Dimethylformamide (DMF) aprotic solvent to get azo pyrimidine substituted pyrazolo[3,4-b]pyridine (5). The azo pyrimidine intermediate (5) proceeds for reduction by palladium catalyst using DMF aprotic solvent to obtain pyrimidine-4,5,6-triamine substituted pyrazolo[3,4-b]pyridine (6). Subsequently, the compound (6) undergoes a mild reaction with methyl chloroformate in a polar protic solvent, resulting in its conversion into a carbamate. This is the final step in the synthesis of the vericiguat drug [28-33].

### 2.2 Structure-activity Relationship of Vericiguat

According to the studies, vericiguat's central skeleton, which is in a purple shade, gives the sGC-stimulating properties and sustained metabolism. Preferable backbone fragments include 1H-indazole, 1H-benzopyrazole, imidazo[1,5-b]pyridazine, and 1H-pyrazolo[3,4-b]pyridine.

The yellow-shaded carbamate moiety is another fragment in this class of compounds that can be altered. It was possible to slightly improve sGC activity by adding a polar functional group in this structure to decrease lipid solubility and fluoro atoms to increase steric hindrance and block metabolic sites. However, modifications had little to no positive effects on lowering clearance. Keeping the terminal methyl group unchanged and avoiding substitution of the carbamate's N atom can slow down metabolism [34]. Here, the outline of SAR is demonstrated in Fig. (**3**).

## 3 DISSOLUTION OF VERICIGUAT

The drug belongs to the BCS class II category. Under fed conditions, the dissolution rate of vericiguat was observed to increase. In a study involving healthy subjects administered with a 10 mg intact tablet, it was found that exposure levels (both AUC and Cmax) increased by approximately 40% when taken with food compared to a fasted state. This substantiates the presence of a food effect on the bioavailability of vericiguat. Furthermore, it was noted that there was a reduction of approximately 20% in interindividual variability in exposure, irrespective of the type of meal consumed. In the case of a 10 mg dose of vericiguat administered *via* intact tablets under fed conditions, the absolute bioavailability was determined to be 93%. Additionally, in healthy subjects administered with vericiguat doses ranging from 2.5 mg to 10 mg *via* intact tablets in a fed state, the drug demonstrated dose proportionality. Dissolution studies indicated no discernible differences between the various formulations, and this was subsequently corroborated through *in vivo* studies.

## 4 PHARMACOKINETICS

Vericiguat has an anticipated pharmacokinetics profile with no extended effects on the blood pressure in patients with left ventricular ejection fraction below 45% and heart failure conditions. Vericiguat's pharmacokinetics have been studied in twenty eight Phase 1 studies (13 clinical DDI studies), three Phase 2 studies, and one Phase 3 study. Furthermore, several populaces Pharmacokinetic (popPK) models for vericiguat were established using the plasma concentration results of clinical experiments. These popPK models have been used to characterize and develop Exposure-Response (ER) relationships in heart failure patients. Furthermore, experimental Physiological based pharmacokinetic modeling and simulation (PBPK) models were built to replicate the predicted pharmacokinetic behavior in phase II patients of HF, as well as the steady-state pharmacokinetics in individuals with severe liver and kidney disorders. Besides that, the applicant conducted a wide range of *in vitro* experiments to identify the enzymes involved in vericiguat metabolism and scrutinize possible enzyme induction, inhibition, and transporter inhibition.

Vericiguat is rapidly absorbed after oral administration, with a tmax of 1-2 hours in a fasting state. When the IR tablet is taken with a meal, the median tmax increases to approximately 4 hours, and the bioavailability of vericiguat also increases. Administration of the 10 mg tablet with food leads to an increase of nearly 40% in the highest concentration (Cmax) and exposure (AUC). Furthermore, taking the tablet with food also results in reduced fluctuation. After administering a 10 mg IR tablet along with either a high carbohydrate meal with low fat or a high calorie with high fat meal, the bioavailability of the drug was nearly equivalent. Vericiguat has a relatively high oral bioavailability of about 93% when carried with food [35]. The same IR tablet formulations have been utilized across vericiguat development without significant changes. As a result, no bioequivalence tests have been carried out. When vericiguat is taken *via* the oral route in the form of a crushed tablet in water has comparable exposure and peak plasma levels to that of an entire dosage form. Consistent pharmacokinetics were detected through the examined dose range during the 7 days of the study (involving single and multiple doses of 0.5 to 15 mg and 1.25 to 10 mg, respectively). Vericiguat has shown dose-proportional pharmacokinetics in healthy individuals and fractionally less than the dose proportionates in disciplines with HFrEF. The pharmacokinetics of vericiguat have seemed to be time-independent. To exemplify, after 7 days of quaque die (once a day) dosing with a terminal half-life of 20-25 h, vericiguat deposit in healthy volunteers was around 150 to 170%. A Vericiguat equilibrium state was accomplished in patients with HFrEF in roughly 6 days, with a terminal half-life of nearly 30 hours.

Vericiguat has a high *in vitro* plasma protein (albumin) binding (97.8%) property. The ratio of blood-to-plasma is 0.656, revealing that red blood cells are not a target of vericiguat. In healthy individuals, the steady state vericiguat volume of distribution is 44 L, which is nearer to the entire amount of body water, implying constrained tissue distribution. For HFrEF subjects, the apparent volume of distribution after oral ingestion of vericiguat with a meal was up to 47 L.

**Fig. (4):**
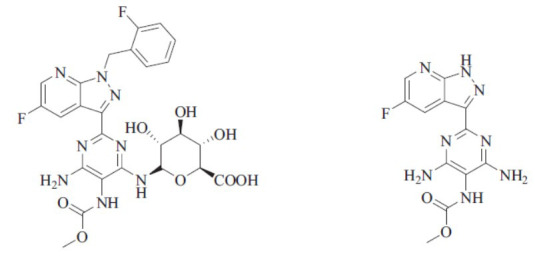
UGT1A1 and UGT1A9 - Metabolites of vericiguat.

Vericiguat is primarily metabolized upon oral ingestion to M-1, an inactive glucuronide metabolite. The parent drug available in plasma is around 28%. The major UGTs associated with complexation are UGT1A1 and UGT1A9 (Fig. **4**), which are found in high concentrations in the hepatic and renal tissues, respectively [36].

**Fig. (5):**
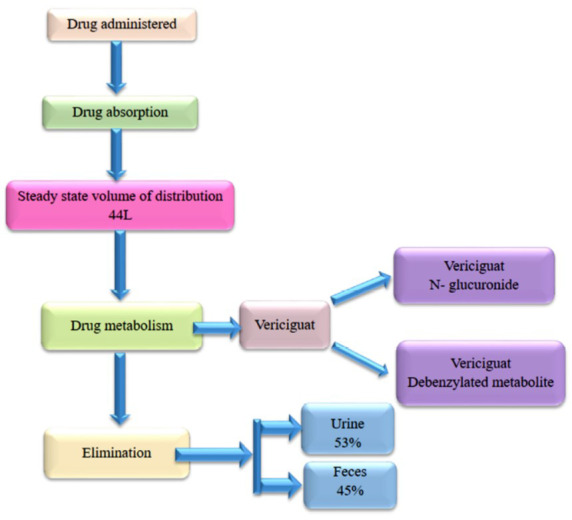
Pharmacokinetic flow of the drug.

According to the ADME analysis, nearly half of the radioactive metabolites are eliminated through urine (53%) [37, 38] and another half *via* feces (45%). Urine excretion is primarily as M-1, but feces removal is as parent vericiguat. Removal in feces is most probably because of M-1 efflux into bile accompanied through deconjugation back to vericiguat by the gut microbiome. It does not reflect an unconsumed portion or straightforward efflux of the parent drug. Even so, the availability of the Fluoro atom in the structure influences metabolic rate (Fig. **5**) [39, 40].

## 5 PHARMACODYNAMICS

**Fig. (6):**
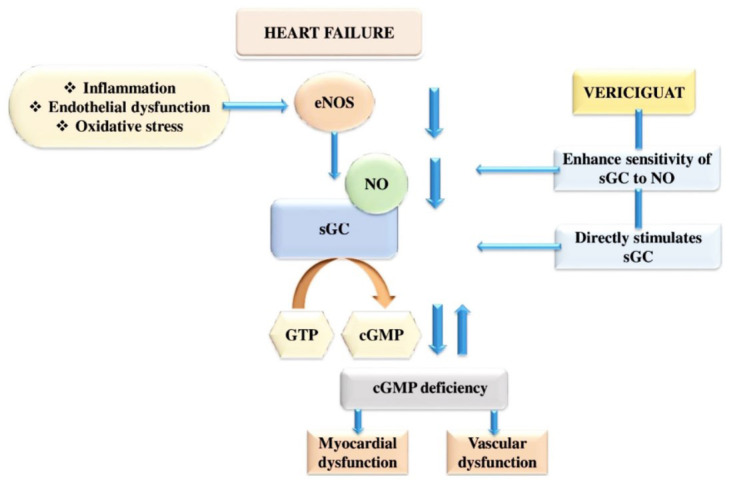
The oxidative stress, inflammation, and endothelial dysfunction conditions of heart failure reduce the eNOS productivity to synthesize vascular NO. In a healthy heart, the synthesized NO interacts with sGC and transforms GTP to cGMP. The cGMP is the ultimate second messenger that is responsible for vascular tonicity, cardiac force, and remodeling. The above pathway gets dysregulated under HF. The vericiguat can directly interact with sGC by activating its β subunit and surpassing the need for NO production through its main pathway, although it brings the same cGMP and its physiological effects.

sGC is a critical enzyme in the nitric oxide (NO) signaling trail [41, 42]. After binding of sGC with NO, it stimulates the production of an intracellular second messenger called cyclic Guanosine Monophosphate (cGMP). The cGMP regulates cardiac contractility, vascular tone, and cardiac remodeling. HF is associated with decreased sGC activity and impaired NO formation, which leads to cardiovascular dysfunction. Vericiguat enhances intracellular cGMP levels through independently activating sGC and in synergism with NO. It results in vasodilation and smooth muscle relaxation (Fig. **6**) [23].

Moreover, sGC is made up of two homologous subunits [43-49]. Each sGC subunit consists of four areas. The first one in the middle is a Per-ARNT-Sim unit, next an N-terminal Haem Nitric Oxide (H-NOX) part, then a catalytic cyclase domain, and the last one is a Coiled-Coil (CC) part. The overall curved CC domain is a compact structure [50]. After binding of NO with haem, the subunit's H-NOX domain and the whole protein stretched partially. The dynamic equilibrium exists amid the lengthy and dormant conditions at this time, so sGC is somewhat triggered. A small amount of guanosine triphosphate is converted to cGMP [51]. cGMP can control cardiac remodeling, vascular tone, and myocardial contractility either directly or indirectly through numerous downstream targets like PDE.

However, it is unclear how cGMP transduces downstream [52-55]. The haem oxidized to a trivalent state leads to sGC shaking off its capability to attach NO. Simultaneously, haem's likeness for sGC is low and easily disrupted. Activators can attach to sGC in the presence or absence of haem and directly activate it. It is especially useful when the sGC activity gets affected by oxidation.

## 6 INFERENCES DRAWN FROM COMPLETED CLINICAL TRIALS

A decade ago, Vericiguat was discovered by Bayer. It is still being tested in multiple clinical trials. To date, 13 clinical trials have been completed. The safety and effectiveness of vericiguat supported based on clinical trial results. Many studies suggest the use of vericiguat as an add-on therapy. vericiguat was found effective in reducing the mortality of high-risk patients from heart failure compared to a placebo. It has been observed that vericiguat reduces the chances of hospitalization, so it indicates that the drug is effective in slowing down the disease progression. At a therapeutic dose, vericiguat has not been found to affect electrolyte balance and kidney functions during clinical trials. Patients with <8000 pg/ml of NT-proBNP get the highest advantage after using the vericiguat. Table **1** indicates the main indications for HF and CAD [56-65].

## 7 PATENT DATA FOR VERICIGUAT DRUG

Patents related to vericiguat hold significant importance not only in protecting intellectual property but also in establishing the safety of this drug for medical use. Through the patenting process, pharmaceutical companies are required to disclose extensive research findings, including data on the drug's safety profile, clinical trials, and manufacturing processes. These publicly available patent documents serve as a valuable database for researchers, healthcare professionals, and regulatory authorities. They provide a comprehensive record of vericiguat's development, safety assessments, and clinical trials, which can be crucial in verifying the drug's safety and efficacy. This transparency fosters trust and confidence in the pharmaceutical industry and regulatory bodies, ensuring that Vericiguat is indeed safe to use for patients suffering from heart failure and related conditions. The following table provides a comprehensive list of patents associated with the pharmaceutical compound vericiguat. These patents encompass various aspects of vericiguat, including its synthesis, medical applications, formulations, and manufacturing processes. Table **2** indicates the patent data for vericiguat drug, including key information on intellectual property and expiration dates [66-78].

## 8 SAFETY AND EFFICACY

Three phase II clinical trials were conducted till 2013. At doses of 2.5-5 mg/10 mg, vericiguat enhanced the excellence of lifespan. It was well tolerated in patients with HFrEF and HFpEF [25, 26]. Even when the amount of dose was enhanced to 15 mg/d, vericiguat demonstrated a slight advantage in terms of developing exercise tolerance (SOCRATES-PRESERVED) [79, 80].

## 9 TOXICOLOGICAL DATA

In repeated dose toxicity of rodent model, changes in hemodynamics, gastrointestinal disturbance, bone deformation, and urinary crystal formation were observed. Ames and micronucleus tests observed the absence of genotoxicity. Carcinogenicity was not observed up to a certain dose level in mice for most of the tissue except pheochromocytoma. There was no reported reproductive toxicity up to the dose of 50 mg/kg/day. Clinically, vericiguat tolerated well at a 50% higher dose (around 15 mg) than its therapeutic dose (2.5 mg to 10 mg). Hypotension may be observed at toxic doses. Due to its high protein binding (98%), dialysis is not useful to detoxify the body [81, 82].

## CONCLUSION

HFrEF is a significant public health concern. In the past several decades, studies on the pathogenesis, prevention, and treatment of HFrEF have produced spectacular outcomes. Nevertheless, after hospitalization, the morbidity as well as mortality rates of the patients are very high [83-85]. Besides, vericiguat may be a promising candidate for heart failure owing to its robust safety and tolerability contour in juveniles as well as aged individuals at all doses in the available literature. Moreover, due to its unique pharmacokinetic and pharmacodynamic profile, vericiguat offers an additional therapeutic advantage. In addition, managing the widening public health issues of HF is a major experiment in which one needs to personalize the treatment of patients in an aging population with limited resources.

## Figures and Tables

**Fig. (1):**
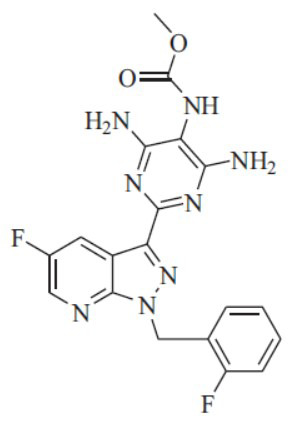
Chemical structure of vericiguat.

**Fig. (2):**
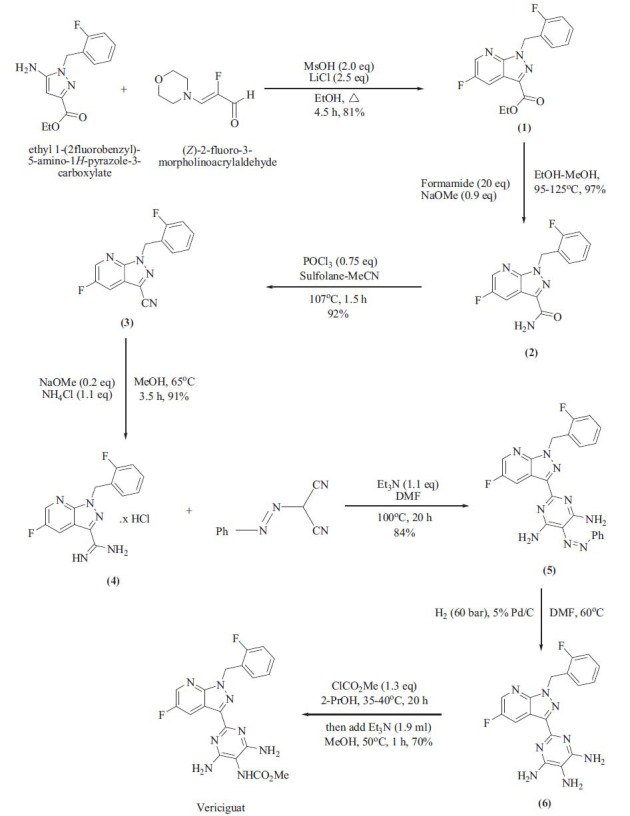
Synthetic pathway for vericiguat.

**Fig. (3):**
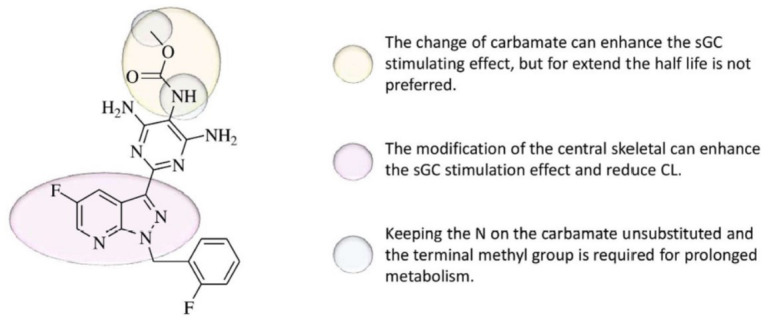
Skeleton of structural activity relationship of vericiguat.

**Table 1:** Clinical trials on vericiguat.

**S. No.**	**Status**	**Timeline**	**Collaborator**	**Type**	**Condition**	**Dose**	**Phase**
1	Recruiting	November 2021	Merk sharp and dohm LLC	Randomized	CHF with reduced ejection fraction (rEF)	2.5,5.0, or 10mg orally once daily	3
2	Not recruiting	August 2022	Merk sharp and dohm LLC	Randomized	Heart failure, Heart failure with rEF	2.5,5 or 10 mg	4
3	Completed	September 2016 to September 2019	Bayer Canadian vigour centre, Duke clinical research institute	Randomized	Heart failure, CHF with rEF	2.5,5,10.0 mg orally once daily	3
4	Completed	May 2017 to October 2017	Bayer	Non-randomized	Pharmacokinetics	10 mg given as 20 x 0.5 mg	1
5	Completed	June 2019 to November 2019	Merck sharp and Dohme LLC Canadian vigour centre Duke clinical research institute	Randomized	CHF with a preserved ejection fraction	2.5,5, or 10 mg orally	2
6	Completed	August 2017 to March 2018	Merk sharp and dohm LLC	Randomized	Coronary artery disease	2.5, 5, 10 mg	1
7	Completed	October 2021 to February 2022	Merk sharp and dohm LLC	Randomized	Heart failure	2.5 or 10 mg	1
8	Completed	November 2015 to August 2016	Merk sharp and dohm LLC	Randomized	Coronary artery disease	2.5 to 10 mg	1
9	Completed	July 2014 to April 2015	Merk sharp and dohm LLC	Non-randomized	Heart failure	2.5 (immediate release tablet)	1
10	Completed	May 2018 to February 2019	Merk sharp and dohm LLC	Randomized	Coronary artery disease	2.5, 5, or 10 mg	1
11	Completed	June 2014 to January 2015	Merk sharp and dohm LLC	Non-randomized	Heart failure	2.5 (for immediate release tablet)	1
12	Completed	November 2013 to June 2015	Bayer	Randomized	Heart failure	1.25 or 5 mg	2
13	Completed	November 2013 to September 2015	Bayer	Randomized	Heart failure	1.25 or 5 mg	2

**Table 2:** Patent data for vericiguat drug.

**S. No.**	**Patent No.**	**Title**	**Publication Date**	**Status**	**Anticipated Expiration**
1	CN108721296B	A kind of compound treats or preventing the purposes in altitude sickness	2019-04-05	Active	2038-07-03
2	TW202042818A	Pharmaceutical composition for the treatment of chronic thromboembolic pulmonary hypertension	2020-12-01	-	-
3	US10844064B2	sGC stimulators	2020-11-24	Active	2035-09-16
4	US10918639	Combination containing sGC stimulators and mineralocorticoid receptor antagonists	2021-02-16	Active	2037-10-05
5	US20210052528A1	The use of sGC activators and sGC stimulators for the treatment of cognitive impairment	2021-02-25	Pending	-
6	US20210177846A1	Use of sGC stimulators for the treatment of mitochondrial disorders	2021-06-17	Pending	-
7	US20210196715A1	Pharmaceutical composition for the treatment of pulmonary arterial hypertension	2021-07-01	Active	2039-12-20
8	US20220031704A1	Novel dual mode of action soluble guanylate cyclase activators and phosphodiesterase inhibitors and uses thereof	2022-02-03	Pending	-
9	EP3411026B1	Use of stimulators of soluble guanylate cyclase for the treatment of nonalcoholic steatohepatitis (nash)	2022-03-09	Active	2037-01-31
10	US11508483	Method of identifying a subgroup of patients suffering from dcSSc that benefits from treatment with sGC stimulators and sGC activators to a higher degree than a control group	2022-11-22	Active	2041-09-22
11	US20230000865A1	Pharmaceutical composition for the treatment of pulmonary vascular disease and/or cardiac dysfunction in Fontan-palliated patients	2023-01-05	Pending	-
12	US20230087609A1	Use of a soluble guanylate cyclase (sGC) stimulator or of a combination of a sGC stimulator and an sGC activator for conditions wherein the heme group of sGC is oxidized or wherein sGC is deficient in heme	2023-03-23	Pending	-
13	WO2023034364A1	Solid state forms of vericiguat and process for preparation thereof	2023-03-09	-	-

## LIST OF ABBREVIATIONS

AUC: Area Under the Plasma Drug Concentration-Time Curve
BCS: Biopharmaceutics Classification System
CAD: Coronary Artery Disease
CC: Coiled-Coil
cGMP: Cyclic Guanosine Monophosphate
CHF: Congestive Heart Failure
Cmax: Maximum Plasma Concentration
DCSSC: Diffuse Cutaneous Systemic Sclerosis
DMF: Dimethylformamide
DMSO: Dimethyl Sulfoxide
eNOS: Endothelial Nitric Oxide Synthase
ER: Exposure-Response
FDA: Food and Drug Administration
GTP: Guanosine Triphosphate
H-NOX: Haem-Nitric Oxide
HF: Heart Failure
HFpEF: Heart Failure with preserved Ejection Fraction
HFrEF: Heart Failure with reduced Ejection Fraction
IR: Immediate Release
LVEF: Left Ventricular Ejection Fraction
NO: Nitric Oxide
NT-proBNP: N-terminal pro-B-type Natriuretic Peptide
PAS: Per-ARNT-Sim
PBPK: Physiologically Based Pharmacokinetic
PDE: Phosphodiesterase
popPK: Population Pharmacokinetics
SAR: Structure-Activity Relationship
sGC: Soluble Guanylate Cyclase
SOCRATES: Soluble Guanylate Cyclase stimulator in Heart Failure patients
TEA: Triethylamine
UGT: Uridine Diphosphate Glucuronosyltransferase
UGT1A1: Uridine Diphosphate Glucuronosyltransferase 1A1
UGT1A9: Uridine Diphosphate Glucuronosyltransferase 1A9
